# Agricultural land use curbs exotic invasion but sustains native plant diversity at intermediate levels

**DOI:** 10.1038/s41598-021-87806-7

**Published:** 2021-04-16

**Authors:** E. Pellegrini, M. Buccheri, F. Martini, F. Boscutti

**Affiliations:** 1 Freshwater Biological Laboratory, Department of Biology, University of Copenhagen, Universitetsparken 4, 3rd floor, 2100 Copenhagen, Denmark; 2 Department of Agricultural, Food, Environmental and Animal Sciences, University of Udine, via delle Scienze 91, 33100 Udine, Italy; 3 Museo Friulano di Storia Naturale di Udine, via Cecilia Gradenigo Sabbadini, 22-32, 33100 Udine, Italy; 4 Trieste, Italy

**Keywords:** Biodiversity, Invasive species

## Abstract

Unveiling the processes driving exotic plant invasion represent a central issue in taking decisions aimed at constraining the loss of biodiversity and related ecosystem services. The invasion success is often linked to anthropogenic land uses and warming due to climate change. We studied the responses of native versus casual and naturalised exotic species richness to land uses and climate at the landscape level, relying on a large floristic survey undertaken in North - Eastern Italy. Both climate and land use drove exotic species richness. Our results suggest that the success of plant invasion at this scale is mainly due to warm climatic conditions and the extent of urban and agricultural land, but with different effects on casual and naturalized exotic species. The occurrence of non-linear trends showed that a small percentage of extensive agricultural land in the landscape may concurrently reduce the number of exotic plant while sustaining native plant diversity. Plant invasion could be potentially limited by land management, mainly focusing on areas with extensive agricultural land use. A more conscientious land management is more and more commonly required by local administrations. According to our results, a shift of intensive to extensive agricultural land, by implementing green infrastructures, seems to be a win–win solution favouring native species while controlling the oversimplification of the flora due to plant invasion.

## Introduction

Biological invasion greatly impacts world biodiversity and ecosystem functionalities and services^1,2^. Invasive plants decrease local species diversity^3^, alter soil biota^4^, increase ecosystem productivity by altering nutrient cycling^5,6^ and impact landscape perception^7^.

Invasive alien species are widely studied in the literature^3,8^ but less is known about potentially future invaders. Exotic casual species and exotic naturalised species are plants that have not reached yet the ultimate “success stage” of the invasion^9^. However, these two categories are extremely important in explaining plant invasion because they represent potentially future invasive species^10^. Casual species are of less concern because their spread is strictly linked to propagule pressure^11^. Instead, naturalised species are well-established species that can shed light on the invasion process even better than invasive plants because they represent the second to last stage of invasion. Predictions of naturalization are expected to be more robust than those for invasiveness and to be less site-specific^12^. In this light, comparing the distributions of casual and native exotics in relation to main environmental drivers might give novel insight into the future invasion scenarios.

The climate was originally proposed as the main filter for plant spread with cold temperatures limiting plant invasion^13,14^. However, the effect of climate on plant invasion is highly scale dependent^15^ and context-dependent^16^. Based on previous findings, invasive species could be favoured by the increase of temperatures due to climate change^17,18^ because of their phenotypic plasticity^19^, but with local environmental conditions affecting significantly this main trend^20^. Invasive species have demonstrated to adapt locally and to quickly evolve during expansion^21^ and ecosystem disturbance was considered more important than climate in the plant invasion process in the Mediterranean basin^20,22^. In fact, climate is fundamental for the progression of the invasion^12^ but exceptions can be explained by site-specific and human related factors, as an effect of an explicit interplay of such factors across the space^15^. Human activities were found to be responsible for enhancing biological invasion especially by increasing propagule pressure^23,24^. Roads, cities, crops and abandoned areas in particular represent primary sources for propagule dispersion^25,26^ because of landscape fragmentation^27^ and habitat vulnerability especially at the ecotone areas^28^. Urbanization is known to cause changes in plant biodiversity because of fragmentation, loss of suitable habitats and increased of pollution^29^. Abandoned agricultural lands remain preferential sites for invasion even after many years from the ceased of agricultural activities^30^. Semi-natural habitats are usually more resistant to plant invasion^23,31^ and a different landscape composition and disturbance seem to determine their degree of invasion^32^. In human-influenced landscapes, exotic species benefit from the increased soil fertility and soil disturbance^14^ but agriculture could also act as a buffer against plant invasion^27,33^. For these reason, the role of agricultural areas in plant invasion has still to be cleared, considering agricultural land use intensity (i.e. rate of crop area vs. linear small patches of semi natural vegetation) as a possible driver of the process.

Plant invasion has been proved to be linked to landscape heterogeneity^27,34^. However, most of the studies on invasive species used fine spatial scales that are seriously affected by biogeographical and historical processes^35,36^. Despite some studies already disentailed the impacts of climate from those attributed to land use^14,33,37,38^, more rarely a distinction is provided between native species, exotic naturalised and exotic casual species. A nice example is provided by Marini et al.^39^ which showed a clear difference in species richness of exotic naturalised species and exotic casual species towards the increase of mean annual temperature.

Therefore, in the present work, the main objectives addressed were (1) to detect the impact of climate and land-use on species richness of exotics and (2) to highlight the possible different responses of exotic casual, exotic naturalised or native species at the landscape scale. As a novelty we considered the effects of different intensity of agricultural land on different categories of exotic species, showing an increasing level of risk in terms of plant invasion (naturalised species > casual species). We expected to find a contrasting response of native versus exotic species and, within exotics, of casual versus naturalised species. In particular, we expect exotics to be favoured by high temperature, precipitation and anthropic land uses cover, with casual exotic more associated to urban land use and roads, considered as main foci of exotic propagules. Moreover, we expected a different impact of intensive and extensive agricultural land use on plant invasion with casual species being less affected by the intensification of agriculture compared to naturalised exotic species. The study area (i.e. Friuli Venezia Giulia, Northern East of Italy) was selected as representing wide ecological gradients in terms of altitude, climate and land use intensity, and on the availability of a solid and updated floristic dataset for the area, useful to test the formulated hypotheses.

## Results

### Species richness

**Figure 1:**
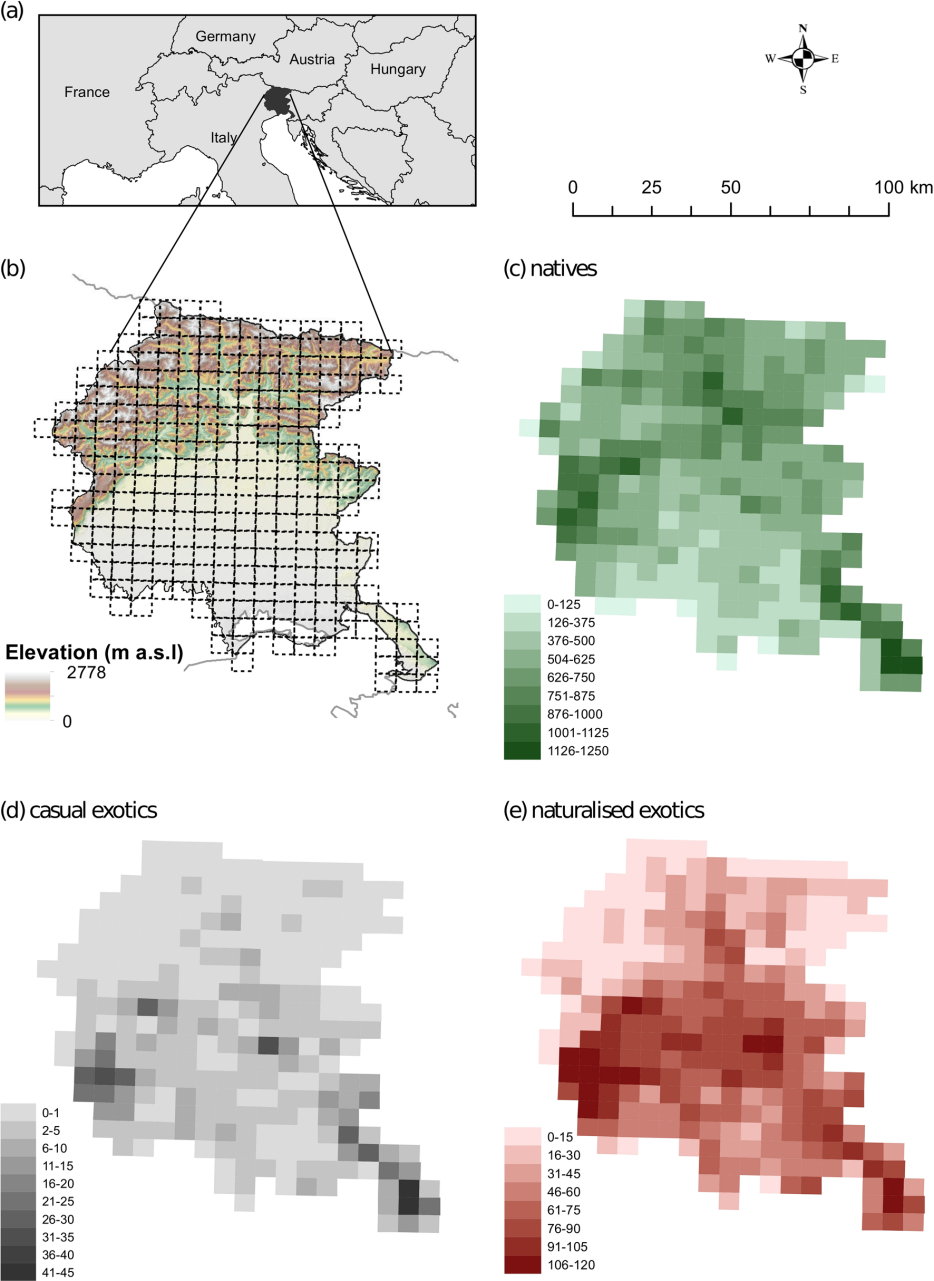
Location of the study area (**a**) and species richness in each grid cell of the three categories of native (**c**), casual exotic (**d**) and naturalised exotic species (**e**). Changes in elevation in the study area and position of grid cells based on the Central European grid for floristic surveys are also reported (**b**). Figure was realised using ArcGIS 10.0 (ESRI).

Alien flora counted 337 species and represented the 15% of the total species (alien plus native) recorded in Friuli Venezia-Giulia (Martini et al. in preparation). Among alien flora, casual (180) and naturalised species (120) were the most abundant, followed by invasive species (37) that represented the 11% of the exotic flora (for further details see^40^). Within each sample area, the number of casual and naturalised species ranged from 0 to 43 (mean ± sd; 4 ± 6) and from 1 to 113 (41 ± 25), respectively, while native species ranges from 150 to 1207 (603 ± 181). Species richness of natives was higher in the Prealp and Karst areas (Fig. 1c), while the richness of naturalised exotic (Fig. 1d) and casual exotic species (Fig. 1e) peaked in the lowlands, Karst area and close to the main towns. Most of the alien species were perennial plants (35% for casual exotic species, 26% for naturalised exotic species), out of these 30% were woody species (trees, shrubs) (17% for casual, 13% for naturalised) and 31% were grasses or forbs (18% for casual, 14% for naturalised). Annual species represent the 38% of the exotic flora, out of these 18% were casual exotic and 20% were naturalised exotic species. The most frequent exotic taxa were *Erigeron annuus* (frequency = 0.89), *Erigeron canadensis* (0.77), *Veronica persica* (0.77), *Robinia pseudoacacia* (0.74), *Helianthus tuberosus* (0.74), *Artemisia verlotiorum* (0.72) and *Oxalis fontana* (0.71).

### Climate and land use as drivers of exotic species richness

**Figure 2:**
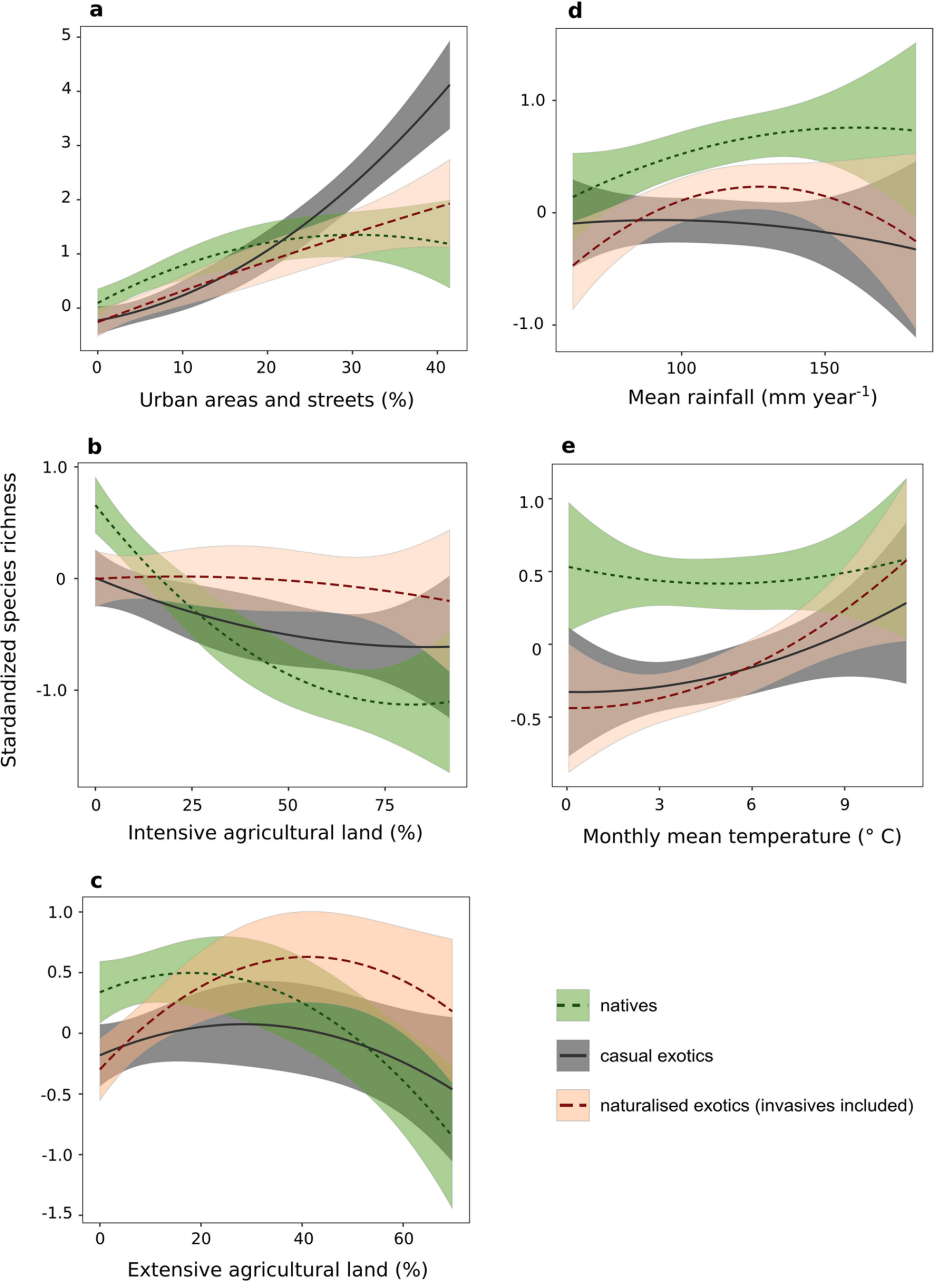
Best selected model using the Multi Model Inference analysis. The model (AIC = 0, R^2^ = 0.69) shows the effects of urban (**a**), intensive and extensive agriculture (**b**, **c**) land uses, annual rainfall (**d**) and mean temperature (**e**) on native (dotted lines) and exotic (casual = solid lines, naturalised = dashed lines) standardised species richness.

Multi Model Inference analysis returned the full model as the only one plausible model (Table 1, Supplementary material Table S1) showing that all climate and land use variables were significant variables explaining species richness of natural, casual and naturalised exotic species (Fig. 2, Supplementary material Table S2). The model explained 69% of the total variation.

**Table 1:** LMMs results obtained for the best selected model after Multi Model Inference analysis. Status referred to natural, casual or naturalised exotic species. Degree of freedom (DF), F value of the statistic (F) and significance level (P) are reported.

	DF	F	P
Status	2, 36	36.00	< 0.001
Extensive agricultural land	2, 36	8.11	< 0.001
Intensive agricultural land	2, 36	14.84	< 0.001
Urban land	2, 36	35.25	< 0.001
Mean rainfall	2, 36	3.41	0.03
Mean temperature	2, 36	2.82	0.06
Extensive agricultural land: status	4, 36	88.06	< 0.001
Intensive agricultural land: status	4, 36	54.93	< 0.001
Urban land: status	4, 36	27.31	< 0.001
Mean rainfall: status	4, 36	4.50	0.002
Mean temperature: status	4, 36	2.84	0.02

Regarding land use, urban areas (including road network) drove the increase of both casual and naturalised exotic species while decreasing native species richness at largest covers (Fig. 2a).

Intensive agricultural land affected negatively both native and casual exotic species richness while exotic naturalised species were almost constant (Fig. 2c).

Extensive agricultural land showed consistent hump-shaped relationships with all considered species pools (Fig. 2e). Nonetheless, species richness of naturalised exotics increased with the increase of extensive agricultural land, with a peak at about 40% presence of extensive agricultural land, while native species showed a maximum value at about 20% of the landscape covered by this land use. Finally, casual species were favoured by intermediate cover of extensive agricultural area (about 30% of cover).

Regarding climate, naturalised exotic species were high at moderate rainfall and high monthly mean temperatures (Fig. 2b,d). Instead, native species increased along with the increase of rainfall and were not substantially influenced by temperature. Casual species follow the trend of naturalised species regarding temperature but were not influenced by rainfall.

## Discussion

Plant invasion is expected to be boosted by climate change^17,20^ and by soil disturbance induced by anthropic land uses^41,42^. In the present work, both climate and land use drove exotic species richness, showing contrasting responses for native species. Our results suggest that the success of plant invasion at the landscape scale is mainly due to the spread of urban and agricultural land use while the occurrence of a small percentage of extensive agriculture (about 20%) may reduce the number of exotic plant and promote native plant diversity.

The percentage of exotic species recorded in the present work is consistent with that reported for Italy, which is about 16%^43^. Except for *Robinia pseudoacacia*, species with larger frequency were herbaceous plants, most of them annuals and belonging to the *Asteraceae* family. This family is the largest among flowering plants^44^ and often associated to most successful invasive species^24^. Annual exotic species has been demonstrated to be highly competitive under anthropogenic disturbance regime^26^. Among the most frequent perennial woody plants many N-fixer species were found (e.g. *R. pseudoacacia, Amorpha fruticosa*), showing to be an impacting life-form^5^, able to change ecosystem proprieties and plant diversity of the invaded ecosystems^6^.

There is no clear agreement among main factors favouring exotic species richness or spread. Some authors strongly support the role of biological traits^45^, phenology^46^ or historical factors about invasion success^47^. Factors affecting plant invasion play an important role at local scale while at large-scale (i.e. landscape), land-use and other large-scale processes (e.g. climate) can hide the local variations of soil and stand structure^48^. Climate and land use were commonly considered as well, but usually focusing on one single species or a specific environment^49,50^.

Precipitation and temperature were already proved to be main driver for plant diversity. It was proposed by the general climate-based hypotheses of plant richness distribution, which theory is based on water-energy dynamics^51,52^. Exotic species were limited in more humid (large mean rainfall) and cold areas, which refer to the alpine and mountain areas^40^, whereas native species were favoured by larger mean rainfall, as proved also for richness of endemic species^53^. Exotic species were found to be related to intermediate precipitation regimes. This behaviour is consistent with the hypotheses that exotic species are particularly sensitive to harsh conditions (e.g. drought stress), preferring intermediate environmental conditions^54,55^.

Literature already demonstrated a contrasting response of native and exotic species in relation to temperature, showing the preference of exotic species for warmer temperatures^39^. The increase in temperature due to climate change is expected to increase exotic species richness and abundance^17^. Upon this view, predictions seem to be more severe into the Mediterranean area, due to a more effective establishment, growth and reproductive rates of exotics compared to the native Mediterranean species^20^. Our study seems to support such expectations, showing that exotic species were more strongly related to temperature rather than natives. However, casual and naturalised exotic species did not show a different pattern towards temperature. In this light, temperature (along with water availability) seems to act as a climatic filter on species, i.e. favouring the naturalization of species of warmer climates. Indeed, we found temperature to be strongly correlated to elevation. For this reason, we can argue about a synergic action of climate and other environmental factors related to elevation (e.g. light intensity, snow cover, nutrients availability) in limiting exotic species, as other studies relating plant invasion to elevation already pointed out^56,57^.

Besides climate, the number of exotic species is also associated with human land management^58^ and plant distribution depends on land use, especially where extreme abiotic gradients are missing^59^. In our dataset, the effect of land use on plant invasion seemed to better explain differences in species richness among native, casual exotic and naturalised exotic species compared to climatic variables, especially considering the shift in the maximum species richness of the three different categories towards extensive agricultural land use. The larger impact on plant invasion of land use, compared to climate, was already reported for the Mediterranean region^20^. Human made environments are more prone to invasion^23,60,61^ and several studies showed that semi-natural habitats are more easily invaded, especially when included in an urban or agricultural context^26,62^.

Cities represent a mosaic of human-made habitats easy to be invaded by exotic species^24,31^. Urban areas are key sites for first introduction and support the propagule pressure effect^63,64^. In our work, casual species were clearly favoured by urban areas and streets, because they have not overcome the reproductive barrier^9^ and their reproductive strategy is restricted on propagule pressure^36,61^. In this process, a particular role is played by main roads^65^, which in our study were included in the urban land use.

We recorded a strong and negative relationship between native species richness and farming (especially intensive farming), whereas the effect on exotic species seemed less strong (casual) or irrelevant (naturalised). Human-managed environments are widely recognised for favouring plant invasion^24,26,61^. Native plant species respond more negatively to agricultural intensification than exotics, probably due to the high soil nutrient availability, that in agricultural area is well represented by phosphorus^66^. Long-term application of chemical fertilizer may promote plant invasion^12^ and have long lasting effects on ecosystems^67,68^. In fact, small patches of natural habitat within the intensive agricultural land have been proved to be the last source of plant diversity in oversimplified agricultural lands but also a focus for weed plants^69,70^.

Intensification of agriculture causes dramatic decline in plant diversity, both to native and exotic species^71^, and affects critically the ecosystem functioning^72^. Nevertheless, at intermediate degree of soil disturbance, there is a positive relation between disturbance and invasion by exotic plants^60^. Anthropogenic disturbance in association with a large heterogeneity of the landscape was reported to promote species richness, of both native and exotic plants^73^. The intermediate disturbance hypothesis is widely recognised and support the idea that intermediate levels of disturbance result in the highest levels of species richness^74,75^. Our work upheld the extension of such hypothesis to landscape disturbance processes, for which the pressure of extensive farming revealed different thresholds for the three different categories analysed, i.e. native, casual exotic or naturalised exotic species. The increasing percentage of extensive agricultural land corresponded to a different peak in species richness of natives (around 20% of extensive agricultural land), casual exotics (30%) and naturalised exotics (40%). Plant invasion has been also proved to be strictly linked to landscape heterogeneity^34^ and our study area comprised a wide range of ecosystems with large differences in climate, ecological conditions and land use. Extensive agricultural land comprises a larger heterogeneity of the landscape compared to the intensive agricultural land, including hedges and grassland sides, differences in land management and type of disturbance. Therefore, the land management on the extent of extensive agriculture, at the regional scale, could be potentially crucial for preserving native species and limiting plant invasion.

## Conclusion

Our work showed that plant invasion is boosted at a landscape scale by increasing temperatures and large land use of extensive agriculture. However, a small area of extensive agriculture improves biodiversity of native plant species. A more conscientious land management is more and more commonly required by local administrations and, from our results, the reduction of extensive agricultural land seems to be a strategic choice for favouring native species while controlling the simplification of the flora due to plant invasion. A landscape perspective is necessary to face the challenge of maintaining productive lands while promoting biodiversity conservation and sustaining ecosystem services^76^. Our study suggests that a trade-off between productive purposes and environmental sustainable schemes might be a win–win solution not solely to sustain biodiversity but also to help the great effort of policies aimed at containing the biological invasion.

## Material and methods

### Study site

The study area was the Friuli Venezia-Giulia region (FVG, centroid coordinates = 46° 24′ 00″ N, 13° 04′ 50″ E, Fig. 1a). It covers an area of about 7900 km^2^ and is bounded by the Julian and Carnic Alps to the north, the Adriatic Sea to the south and the Karst of Trieste to the east. FVG exhibits a high geological and morphological complexity^77^, showing a prevalence of calcareous soils (i.e. limestone and dolomites) in the mountain region, alluvial calcareous deposits in the lowlands and the occurrence of both rock and sandy coasts^77^. FVG exhibits a continental climate in the inner valleys and temperate conditions in the outer area, with a mean annual air temperature of about 12 °C. Due to its ecological complexity, FVG has been recognised for its considerable plant diversity since early botanical surveys^78^. The area encompasses several protected areas including well-conserved wetlands, grasslands and ancient woods. Landscape variability determines a large complexity of land uses, mainly dedicated to the intensive agriculture in the plain. In these cultivated areas both annual (maize, barley, soybean) and perennial crops (vineyards, orchards, poplar) are frequent^79^. FVG was selected as study area because of the occurrence of clear altitudinal and climatic gradients, ranging from the coast to the Alps and the strong impact of human land uses. Moreover, an intensive and updated floristic survey was conducted during the last 40 years resulting in a solid database of presence and location of native or exotic species (see below).

### Plant species distribution

In the present study, FVG was divided in 273 cells (Fig. 1b) based on the Central European grid for floristic surveys^80^. Each grid cell was about 3′ of latitude × 5′ of longitude (ca. 5.5 × 6.5 km). Species distribution data relied on an ongoing survey aimed at creating a new vascular plant atlas for the FVG region (data from Martini F. and collaborators, for details see Acknowledgment section). The dataset has more than 290,000 records, including field data, herbarium specimens and literature data (period 1980–2020). The data were collected during a coordinated field sampling programme providing comparable and sufficient sampling effort in all the cells of the region (Supplementary material Fig. S1). The sampling effort in the different cells was related to the saturation level of the species accumulation curves, i.e. a cell was explored until the number of species tended to saturation.

The occurrence of taxa was recorded for each grid cell. Species were divided into 3 groups: native, casual exotic and naturalised exotic species. Naturalised exotic species included invasive species. Following the accepted framework for biological invasion^9^, a casual species overtakes the survival barrier whereas a naturalised species overcomes the following barriers of reproduction and dispersal. Nomenclature and taxonomy followed the Italian flora check-lists^43,81^. Exotic status (i.e. casual and naturalised species) was classified according to the regional check-list^40^. For each group, species richness was calculated for a total of 196 cells, excluding those at the border of the area showing incomplete data.

### Environmental predictors

For each of the 196 cells, we calculated 7 environmental variables divided in geomorphological, climatic and land use predictors. Environmental data are reported in Table 2.

**Table 2:** Minimum (min), mean and maximum (max) values per cell grid for elevation, climate and land use data.

	Min	Mean	Max
**Geomorphology**			
Elevation (m)	0	529	1779
**Climate**			
Monthly mean temperature (°C)	0.06	6.07	11.00
Mean rainfall (mm year^−1^)	620	962	1817
**Land use**			
Natural areas (%)	0	47	100
Urban areas and streets (%)	0	7	41
Intensive agricultural land (%)	0	21	91
Extensive agricultural land (%)	0	13	69

The geomorphological predictor selected was the elevation (m a.m.s.l.). Data were obtained from the Digital Elevation Model (DEM) of FVG based on 10 m resolution (IRDAT FVG, http://irdat.regione.fvg.it/). Elevation was in average 529 m a.m.s.l. ranging from 0 to 2,780 m (Coglians mountain).

Climatic data were obtained from the official climatic institute OSMER ARPA (https://www.osmer.fvg.it/) from a grid dataset of 500 m resolution. The mean monthly temperature (°C) per grid cell refers to the annual average of mean monthly temperature in the period 1991–2010 and mean annual rainfall (mm year^−1^) refers to the period 1961–2010. Monthly mean temperature was in average 6 °C and annual mean rainfall was 962 mm.

Land use types were obtained from FVG Land Use cartography based on the maps derived by MOLAND project (1:25,000)^82^ and further updated and modified for ecological networking purposes (http://irdat.regione.fvg.it/WebGIS/). Land use types were merged in 4 different categories: natural areas, urban areas and streets, intensive agricultural land and extensive agricultural land. Each category was expressed as the % of surface occupied by a specific land use inside each grid cell. Natural areas included grasslands, woods and rivers. Urban areas and roads included cities and industrial areas and main road network. The agricultural areas were further divided in two categories, namely intensive and extensive agricultural land use, according to the rate between cropland (arable filed areas) and patches of linear and small semi-natural habitats not detected by scale map (i.e. ditches, grass strips, meadows, hedgerows) (Supplementary material Fig. S2). The mean crop cover within intensive versus extensive agricultural land use was preliminary assessed on 15 circular sample areas in agricultural landscapes of ca. 7.1 km^2^ (radius = 1.5 km) where a detailed maps of ditches, grass strips, meadows, hedgerows was quantified. Intensive agricultural land was largely covered by annual and perennial crops (cropland mean ± sd; 95.1 ± 4.0%) in very simplified crop rotations. Extensive agricultural land use included croplands (66.8 ± 13.8%) but also relevant patchworks of linear or small semi-natural habitats not detected by scale map (i.e. ditches, grass strips, meadows, hedgerows). Average values of variables were calculated for each grid cell using QGIS 2.18^83^.

Considering the whole cell grid, urban land cover was about 7% of each cell area on average. Extensive agricultural land was in average 13%, reaching up to 69% of cover per cell. Intensive agricultural land was higher compared to extensive agricultural land, in average 21% and up to 91%.

### Data analysis

The influence of the considered environmental variables on species richness of native, casual exotic or naturalised exotic species was evaluated with a Multi-Model Inference approach. Prior to modelling, variables were examined for collinearity^84^ by Pearson’s correlation test and by variance inflation factor using the ‘vif’ function^85^. Elevation was removed from the model because highly negatively correlated with the mean temperature (r = − 0.79, *p* < 0.001). Natural areas were not included in the model because complementary to the other land use types (Supplementary material Fig. S3).

We used Linear Mixed-effects Models (LMMs) to estimate model parameters. The model included species richness as response variable, the status (native, casual exotic or naturalised exotic species) as factor, climate and land use predictors as fixed effects. As the number of species of each exotic status (i.e. native, casual and naturalised exotic species) were quantified in the same cells, we included the id of sampling cell as a random factor to correct the number of degrees of freedom. We accounted for possible spatial correlation of data by including in the model the following semivariogram functions: exponential, Gaussian, linear, rational quadratic, and spherical. We used this method as one of the most robust application in ecological data^86^, all the used functions are detailed in Pinheiro and Bates^87^. We fitted the full model and compared all the possible combinations of semivariogram function and selected the best model using the Akaike Information Criterion (AIC). The model considering the rational semivariogram function was the best solution and hence considered in the further model selection.

The Multi-Model Inference analysis was carried-out using R statistical software^88^ with the ‘MuMIn’ package^89^. The LMMs were applied using the “nlme” package^90^. The analysis was carried out on a single model exploring the combined effect of species status and environmental variables.

For each variable, a quadratic term was included in order to consider possible non-linear responses. Within each status level (i.e. native, casual exotic, naturalised exotic), we standardised species richness using the Z standardization function.

Model assumptions were verified looking at diagnostic plot of the distribution of the residues. We used Akaike’s information criterion (AIC) to select the best model (lowest ΔAIC) among all plausible models (ΔAIC < 2)^91^.

## Supplementary Information

Supplementary Information.

## Data Availability

Authors declare that data will archived in a suitable and free repository according to the journal policy.
