# Supplementary material for: Agricultural land use curbs exotic invasion but sustains native plant diversity at intermediate levels
Source: Sci Rep. 2021 Apr 16;11:8385. doi: 10.1038/s41598-021-87806-7 (PMC8052428; doi:10.1038/s41598-021-87806-7)
Supplement: Supplementary file 1 — Supplementary Information. [file 41598_2021_87806_MOESM1_ESM.docx]

**Agricultural land use curbs exotic invasion but sustains native plant diversity at intermediate levels**

Pellegrini E.^12^*, Buccheri M.^3^, Martini F.^4^, Boscutti F.^2^

^1^ Department of Biology, University of Copenhagen, Universitetsparken 4, 3rd floor, 2100 København Ø, Denmark

^2^ Department of Agricultural, Food, Environmental and Animal Sciences, University of Udine, via delle Scienze 91, 33100 Udine, Italy

^3^ Museo Friulano di Storia Naturale di Udine, via Cecilia Gradenigo Sabbadini, 22-32, 33100 Udine, Italy

^4^ via Fortunio 10, I-34141 Trieste, Italy

**Supplementary material**

Fig. S1. Number of records (a) and survey effort (b) per grid cell in the study area. A cell was explored until the number of species tended to saturation.


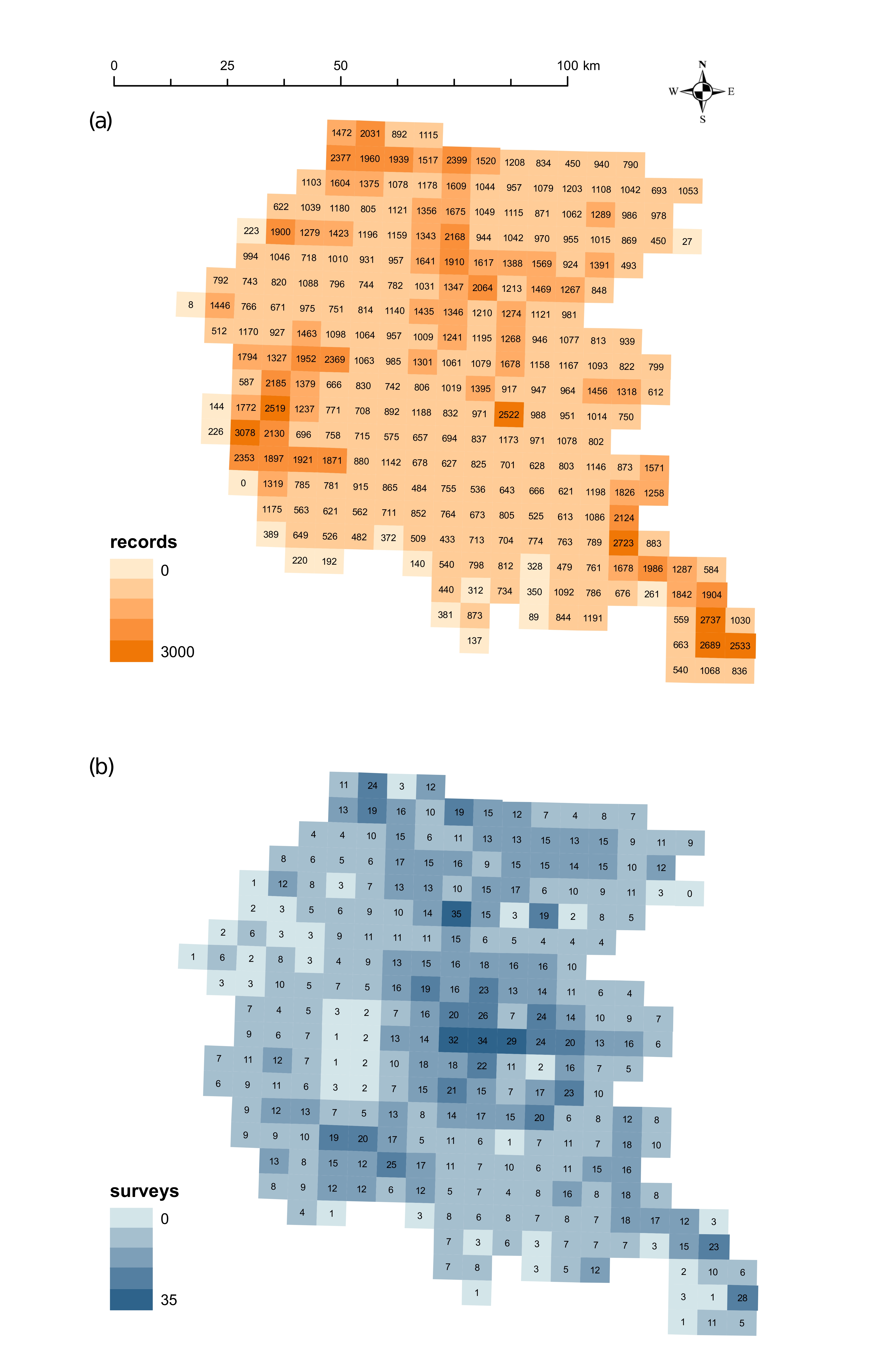


Fig. S2. Example of habitat selection for the definition of the three categories considered for the land use, i.e. urban areas and streets, extensive agricultural land and intensive agricultural land. Patches not coloured (background aerial photo) refer to natural areas. Figure was realised using ArcGIS 10.0 (ESRI).


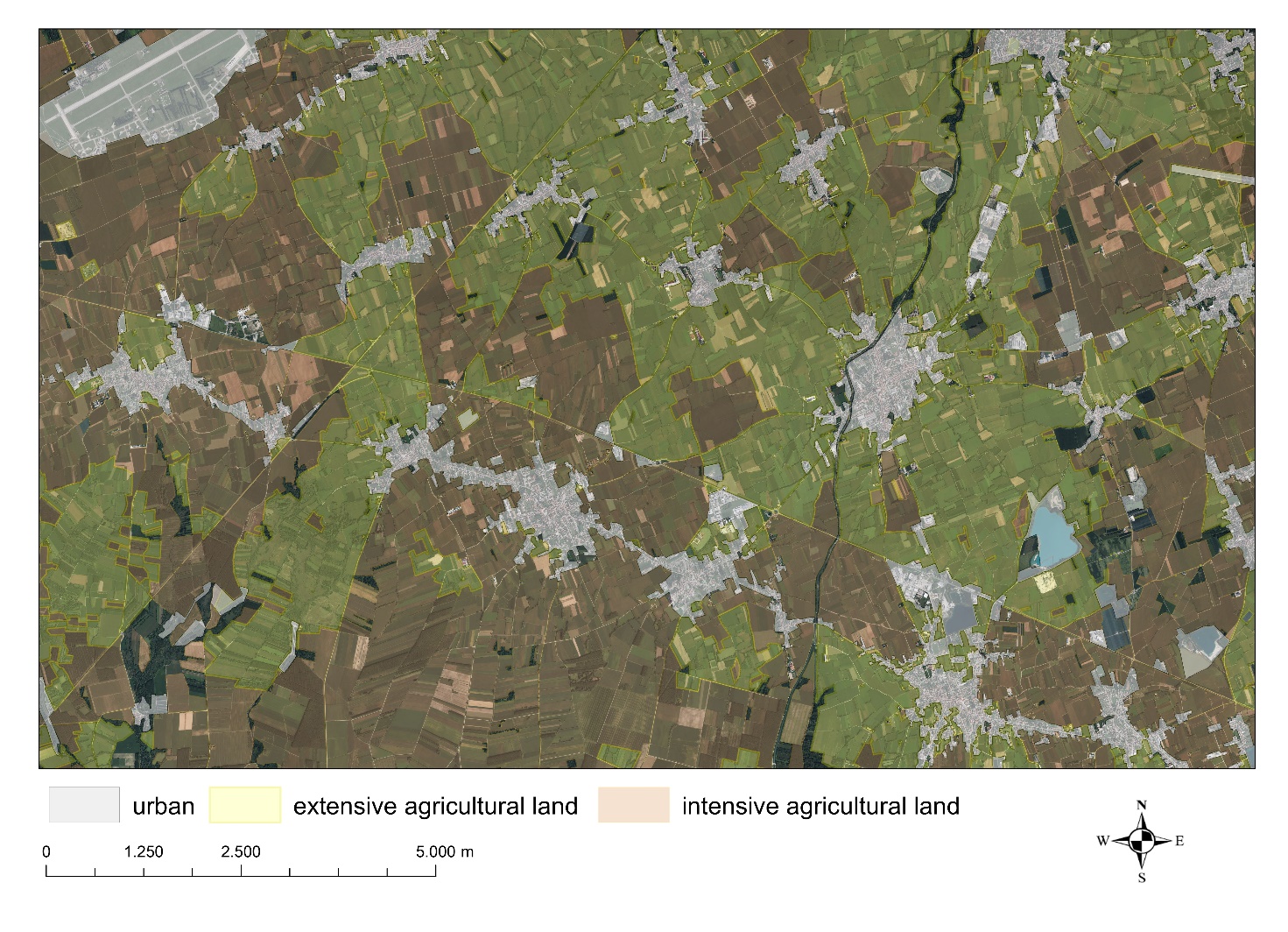


Fig. S3. Correlation plot between independent variables of climate and land use. Elevation was removed from modelling because highly correlated to monthly mean temperature. Natural areas were also removed because complementary to the other land uses.


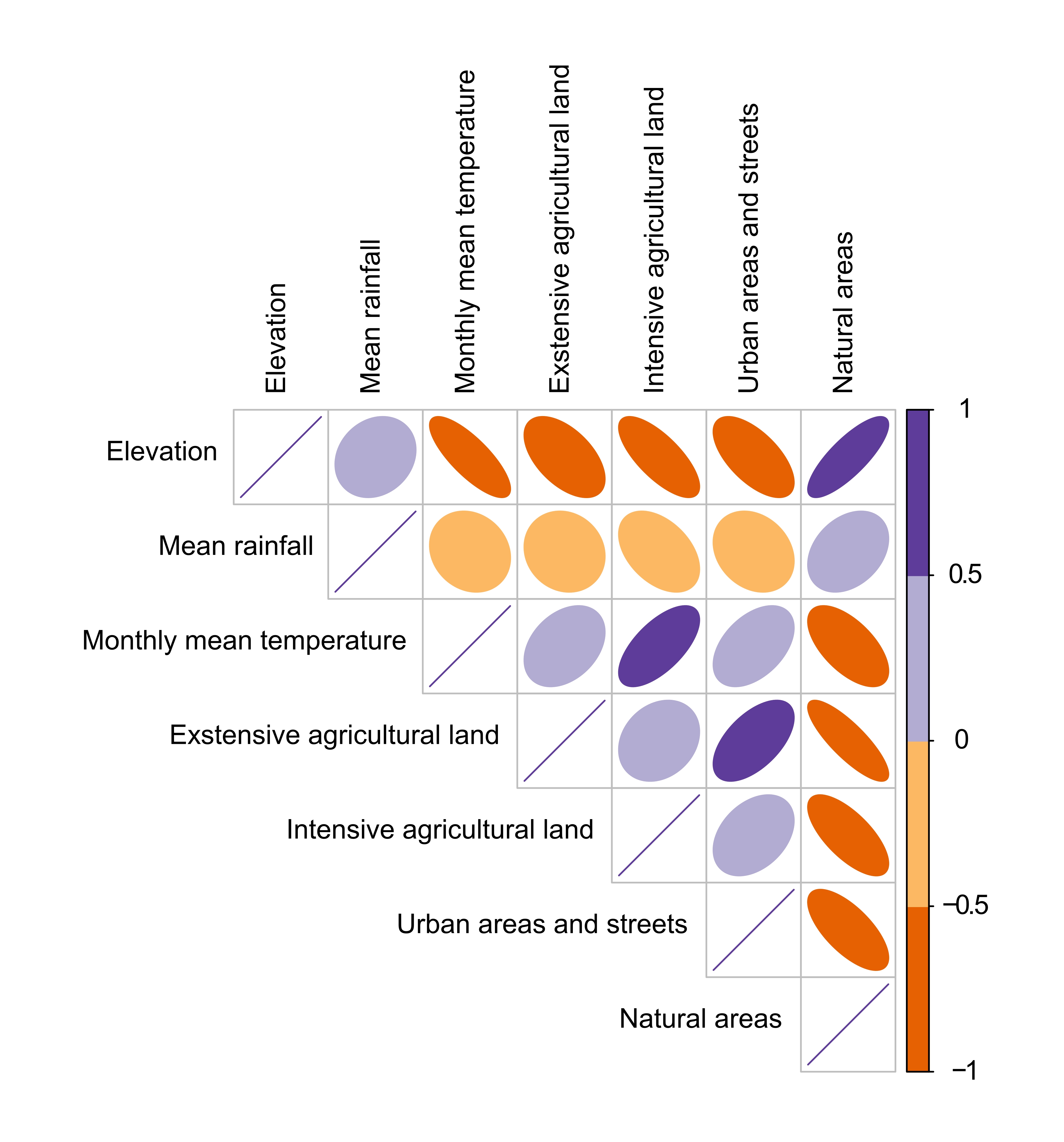


Table S1. First ten models and null model (italics) obtained from the multi-model inference analysis and ranked according to their AIC values. Estimates indicated the intercept, the variables considered in each model (mean rainfall, monthly mean temperature, extensive agricultural land use, intensive agricultural land use, urban areas and stress) including a quadratic term (variable, 2) and with the interactions with the species status (native, casual or naturalised species). Model R^2^, degrees of freedom (df), AIC, ΔAIC, and model weight are also shown.

| **Model ID** | ***Intercept*** | **Poly (mean rainfall, 2)** | **Poly (extensive agricultural land, 2)** | **Poly (intensive agricultural land, 2)** | **Poly (urban areas and streets, 2)** | **Poly (monthly mean temperature, 2)** | **Status** | **Poly (mean rainfall, 2) : status** | **Poly (extensive agricultural land, 2) : status** | **Poly (intensive agricultural land, 2) : status** | **Poly (urban areas and streets, 2) : status** | **Poly (monthly mean temperature, 2) : status** | **R^2^** | **df** | **AIC** | **∆ AIC** | **Weight** |
| --- | --- | --- | --- | --- | --- | --- | --- | --- | --- | --- | --- | --- | --- | --- | --- | --- | --- |
| 2048 | -0.05 | + | + | + | + | + | + | + | + | + | + | + | 0.69 | 35 | 932.3 | 0.0 | 0.999 |
| 1024 | -0.05 | + | + | + | + | + | + | + | + | + | + |  | 0.68 | 31 | 946.0 | 16.7 | 0.001 |
| 1984 | -0.05 | + | + | + | + | + | + |  | + | + | + | + | 0.68 | 31 | 948.6 | 16.2 | 0.000 |
| 1008 | -0.05 | + | + | + | + |  | + | + | + | + | + |  | 0.68 | 29 | 951.9 | 19.6 | 0.000 |
| 1983 | -0.05 |  | + | + | + | + | + |  | + | + | + | + | 0.67 | 29 | 953.2 | 20.8 | 0.000 |
| 960 | -0.05 | + | + | + | + | + | + |  | + | + | + |  | 0.67 | 27 | 961.1 | 28.8 | 0.000 |
| 959 | -0.05 |  | + | + | + | + | + |  | + | + | + |  | 0.67 | 25 | 965.7 | 33.4 | 0.000 |
| 944 | -0.05 | + | + | + | + |  | + |  | + | + | + |  | 0.67 | 25 | 967.0 | 34.6 | 0.000 |
| 1920 | -0.05 | + | + | + | + | + | + | + | + |  | + | + | 0.66 | 31 | 970.9 | 38.5 | 0.000 |
| 943 | -0.05 |  | + | + | + |  | + |  | + | + | + |  | 0.66 | 23 | 972.3 | 40.0 | 0.000 |
| *1* | *0.04* |  |  |  |  |  |  |  |  |  |  |  | *0.15* | *3* | *1505.3* | *573.0* | *0.000* |

Table S2. Summary of the best model obtained from the multi-model inference analysis showing estimates, degrees of freedom (df) and significance of each coefficient of the model. The poly() term refers to a polynomial function applied to the variable. Interactions between independent variables and species category (natives, casual exotics and naturalised exotics) are also shown.

|  | **Estimate** | **df** | ***P value*** |  |
| --- | --- | --- | --- | --- |
| *Intercept* | -0.05 | 360 | 0.248 |  |
| Poly(extensive agricultural land, 2)1 | 0.78 | 360 | 0.681 |  |
| Poly(extensive agricultural land, 2)2 | -2.36 | 360 | 0.002 | * |
| natives | 0.12 | 360 | 0.002 | * |
| naturalised | 0.14 | 360 | <0.001 | * |
| Poly(intensive agricultural land, 2)1 | -5.76 | 360 | 0.005 | * |
| Poly(intensive agricultural land, 2)2 | 1.02 | 360 | 0.514 |  |
| Poly(urban areas and streets, 2)1 | 14.17 | 360 | <0.001 | * |
| Poly(urban areas and streets, 2)2 | 4.21 | 360 | 0.005 | * |
| Poly(mean rainfall, 2)1 | -0.55 | 360 | 0.666 |  |
| Poly(mean rainfall, 2)2 | -0.51 | 360 | 0.663 |  |
| Poly(monthly mean temperature, 2)1 | 3.37 | 360 | 0.037 | * |
| Poly(monthly mean temperature, 2)2 | 0.81 | 360 | 0.497 |  |
| Poly(extensive agricultural land, 2)1: natives | -3.66 | 360 | 0.034 | * |
| Poly(extensive agricultural land, 2)2: natives | -1.38 | 360 | 0.294 |  |
| Poly(extensive agricultural land, 2)1: naturalised | 5.94 | 360 | 0.001 | * |
| Poly(extensive agricultural land, 2)2: naturalised | -1.74 | 360 | 0.185 |  |
| Natives: poly(intensive agricultural land, 2)1 | -11.17 | 360 | <0.001 | * |
| Naturalised: poly(intensive agricultural land, 2)1 | 5.11 | 360 | 0.006 | * |
| Natives: poly(intensive agricultural land, 2)2 | 2.25 | 360 | 0.112 |  |
| Naturalised: poly(intensive agricultural land, 2)2 | -1.57 | 360 | 0.268 |  |
| Natives: poly(urban areas and streets, 2)1 | -5.70 | 360 | <0.001 | * |
| Naturalised: poly(urban areas and streets, 2)1 | -4.11 | 360 | 0.011 | * |
| Natives: poly(urban areas and streets, 2)2 | -7.28 | 360 | <0.001 | * |
| Naturalised: poly(urban areas and streets, 2)2 | -4.55 | 360 | 0.001 | * |
| Natives: poly(mean rainfall, 2)1 | 3.87 | 360 | 0.001 | * |
| Naturalised: poly(mean rainfall, 2)1 | 3.59 | 360 | 0.002 | * |
| Natives: poly(mean rainfall, 2)2 | -0.47 | 360 | 0.653 |  |
| Naturalised: poly(mean rainfall, 2)2 | -2.05 | 360 | 0.052 | . |
| Natives: poly(monthly mean temperature, 2)1 | -3.12 | 360 | 0.032 | * |
| Naturalised: poly(monthly mean temperature, 2)1 | 2.23 | 360 | 0.124 |  |
| Natives: poly(monthly mean temperature, 2)2 | -0.11 | 360 | 0.914 |  |
| Naturalised: poly(monthly mean temperature, 2)2 | 0.48 | 360 | 0.651 |  |
